# The Disruption of *Cyp7b1* Controls IGFBP2 and Prediabetes Exerted Through Different Hydroxycholesterol Metabolites

**DOI:** 10.3390/ijms262411994

**Published:** 2025-12-12

**Authors:** Roberto Martínez-Beamonte, Natalia Guillén, Javier Sánchez-Marco, Luis V. Herrera-Marcos, Joaquín C. Surra, María A. Navarro, Cristina Barranquero, Carmen Arnal, Juan J. Puente, Ma Jesús Rodríguez-Yoldi, Isabel Mendiara, Celia Domeño, Cristina Nerín, Aron M. Geurts, Jesús Osada, Martín Laclaustra

**Affiliations:** 1 Departamento de Bioquímica y Biología Molecular y Celular, Facultad de Veterinaria, Instituto de Investigación Sanitaria de Aragón, Universidad de Zaragoza, E-50013 Zaragoza, Spain; romartin@unizar.es (R.M.-B.); nguillen@unizar.es (N.G.); javiersanchezmarc@gmail.com (J.S.-M.); lherrera@unizar.es (L.V.H.-M.); angelesn@unizar.es (M.A.N.); cbarranquero.iacs@aragon.es (C.B.); 2 Instituto Agroalimentario de Aragón, CITA, Universidad de Zaragoza, E-50013 Zaragoza, Spain; jsurra@unizar.es (J.C.S.); mjrodyol@unizar.es (M.J.R.-Y.); 3 CIBER de Fisiopatología de la Obesidad y Nutrición, Instituto de Salud Carlos III, E-28029 Madrid, Spain; 4 Departamento de Producción Animal y Ciencia de los Alimentos, Escuela Politécnica Superior de Huesca, Instituto de Investigación Sanitaria de Aragón, Universidad de Zaragoza, E-22002 Huesca, Spain; 5 Departamento de Patología Animal, Facultad de Veterinaria, Universidad de Zaragoza, E-50013 Zaragoza, Spain; arnal@unizar.es; 6 Servicio de Bioquímica Clínica, Hospital Clínico Universitario Lozano Blesa, E-50009 Zaragoza, Spain; jjpuente@salud.aragon.es; 7 Departamento de Farmacología, Fisiología, Medicina Legal y Forense, Instituto de Investigación Sanitaria de Aragón, Universidad de Zaragoza, E-50013 Zaragoza, Spain; 8 Instituto de Investigación en Ingeniería de Aragón (I3A), Universidad de Zaragoza, E-50018 Zaragoza, Spain; isabel.mendiara@gmail.com (I.M.); cdomeno@unizar.es (C.D.); cnerin@unizar.es (C.N.); 9 Human Molecular Genetics Center, Medical College of Wisconsin, Milwaukee, WI 53226, USA; ageurts@mcw.edu; 10 Departamento de Medicina, Facultad de Medicina, Instituto de Investigación Sanitaria de Aragón, Universidad de Zaragoza, E-50009 Zaragoza, Spain; mlaclaus@unizar.es; 11 CIBER de Enfermedades Cardiovasculares, Instituto de Salud Carlos III, E-28029 Madrid, Spain

**Keywords:** CYP7B1, rat, HDL, IGFBP2, LDLR, 25-hydroxycholesterol

## Abstract

Cytochrome P450, family 7, subfamily b, polypeptide 1 (CYP7B1) is a widely expressed enzyme involved in the hydroxylation of sterols. Generated by transposon technology in zygotes, male rats lacking *Cyp7b1* expression in homozygosis showed an absence of *Cyp7b1* mRNA expression in the liver, small intestine, adipose tissue, and muscle. Elevated levels of 25-hydroxycholesterol were found in the liver of mutant rats. After overnight fasting, plasma triglyceride (TG) levels were increased in the homozygous rats. In agreement with this, increased hepatic secretion of very-low-density lipoprotein-TG (VLDL) in fasting rats treated with tyloxapol and decreased low-density receptor protein (LDLr) on the hepatocyte plasma membranes were observed. The decrease in LDLr was not due to decreased mRNA expression but to increased expressions of its proteases (*Psck9* and *Mylip*). RNA sequencing identified *Fasn*, *Igfbp2*, and *Pcsk9* as targets of the *Cyp7b1* absence. However, the hepatic protein contents of IGFBP2 were increased in *Cyp7b1*-deficient rats, accompanied by a normal glucose tolerance test. HepG2 cells lacking *CYP7B1* showed increased expressions of *FASN* and *IGFBP2*. These results suggest a role of CYP7B1 in the control of hepatic IGFBP2 and VLDL-TG secretion as a prediabetes sign exerted through 25-hydroxycholesterol and transcriptional or translational mechanisms depending on the species.

## 1. Introduction

Cytochrome P450, family 7, subfamily b, polypeptide 1 (CYP7B1) is a microsomal enzyme involved in the alternative route of bile acid biosynthesis by converting 27-hydroxycholesterol into 5-cholesten-3β,7α,27-triol (reviewed in [1,2]). This enzyme also hydroxylates 25-hydroxycholesterol, 26-hydroxycholesterol, and 3β-hydroxy-5-cholenoic acid (CA27-∆5-3β-ol) at carbon 7 (reviewed in [3,4]). A role in NADPH-dependent 7alpha-hydroxylation of androstenediol, dehydroepiandrosterone, pregnenolone, and other steroids has also been proposed [5]. In agreement with these functions, mice lacking Cyp7b1 showed increased plasma, kidney, and liver levels of 25- and 27-hydroxycholesterol [6] and an inability to convert dehydroepiandrosterone into its metabolite in several organs such as the brain, spleen, heart, prostate, lung, and ovary [7]. Since CYP7B1-mediated 7alpha-hydroxylation abolishes the estrogen receptor alpha and beta-stimulating effect of 5-androstene-3beta,17beta-diol and 5alpha-androstane-3beta,17beta-diol [8], female Cyp7b1-deficient mice experienced continuous estrogenic stimulation of mammary glands and uteri [9]. Likewise, the prostates of male mice were hypoproliferative before puberty and smaller after puberty than those of wild-type mice [10]. Thus, CYP7B1 is a critical extrahepatic hydroxylase that may protect tissues from oxysterol toxicity and is also expressed in tissues involved in bile acid absorption, such as the small intestine and colon [11]. Furthermore, in the brain, liver, and skin, dehydroepiandrosterone (DHEA) is converted by CYP7B1 to 7α-hydroxy-DHEA [12]. The latter is a substrate for 11β-hydroxysteroid dehydrogenase type 1, which also converts inactive cortisone into active cortisol. In this sense, high levels of 7α-hydroxy-DHEA compete with the enzyme and may affect the availability of active cortisol. Therefore, CYP7B1 indirectly modulates cortisol levels in these tissues [13]. Given this broad range of actions, patients with mutations in CYP7B1 show spastic paraplegia type 5 with great variability in clinical presentation and disease course [14,15]. CYP7B1 loss has been proposed as an early alteration in the course of insulin resistance and may be related to the progression of the syndrome from insulin resistance to type 2-like diabetes [16] and other liver pathologies [3].

27-hydroxycholesterol (27-HC), which accumulates in CYP7B1 deficiency, has been shown to be an endogenous ligand for liver X receptors α and β in cholesterol-loaded cells [17], and these receptors participate in cholesterol and triglyceride biosynthesis and are modulators of high-density lipoprotein (HDL) functions by activating ABC transporters [18]. The relevance of these tissue culture findings has been challenged in vivo by the absence of cholesterol changes in Cyp7b1-deficient mice [6]. Furthermore, 27-HC has been found to be an endogenous selective estrogen receptor modulator in vascular cells and responsible for the loss of estrogen action [18]. 25-hydroxycholesterol (25-HC) is involved in local and systemic immune responses [19] and contributes to nervous system diseases, macular degeneration, atherosclerosis, and cancer development [20].

Rats are common laboratory animals with a long experimental tradition dating back to 1856 [21]. They have in common with the mouse that they are HDL mammals because, unlike humans, these are the predominant lipoproteins in their plasma due to the absence of cholesterol ester transfer protein [22] and they secrete APOB48 from the liver [23]. This animal shows increased metabolism of cholesterol to bile acids when fed increased dietary cholesterol [24] and is also considered more reliable than the mouse in the study of diabetes and related conditions [25]. Its genome size of 2.8 Gb and 22 chromosomes is a step forward to be compared with the human one (3.1 Gb genome size and 24 chromosomes) [26]. These facts let us hypothesize that the absence of CYP7B1 in rats may be translated in diabetes or prediabetes alterations. To this end, rats lacking CYP7B1 were generated by transposon insertion and we characterized their glucose and lipid metabolism. They showed increased hepatic levels of 25-HC and plasma hypertriglyceridemia after an overnight fast. This finding was consistent with abnormal VLDL-TG secretion without an alteration in glucose tolerance. Using RNA sequencing, a protein related to insulin, IGFBP2, was identified as a target of this gene inactivation. These findings suggest a role for CYP7B1 in VLDL-TG, a sign of insulin resistance, as well as in the regulation of IGFBP2.

## 2. Results

### 2.1. Modification of the Cyp7b1 Gene in Rat Zygotes

Supplementary Figure S1 illustrates the strategy for targeting the *Cyp7b1* gene using the Sleeping Beauty transposon BART3 gene-trap system in Fischer 344 inbred background rats. One transposon was inserted into the first intron of this gene (Supplementary Figure S1A). Using specific primers for the region and the transposon, it was shown to produce a 538-bp-targeted allele in homozygous and heterozygous animals (Supplementary Figure S1B). When RT-PCR was used to amplify a fragment of 101-bp corresponding to exons 3 and 4 of mature *Cyp7b1* mRNA, it was observed in the liver, duodenum, jejunum, ileum, visceral fat, subcutaneous adipose tissue, and hind limb (Supplementary Figure S1C,D). The gene-trap strategy resulted in no expression of the *Cyp7b1* transcript in any of studied tissues (Supplementary Figure S1C,D). Using RNAseq (Supplementary Figure S1E), an initiation of transcription was observed in KO rats that was halted at the position of the transposon and as a consequence, neither mRNA was detected and nor was protein observed in the liver (Supplementary Figure S1F).

Homozygous rats carrying the modified *Cyp7b1* gene were healthy, and no abnormality in their development was observed (body weight [253 ± 19 and 277 ± 32 g] and liver weight [8.8 ± 0.5 and 9.3 ± 1 g] for wild-type and mutant rats, respectively); some survived more than 2 years. Both sexes were fertile without differences in the offspring number when comparing mating from heterozygous and homozygous females. However, a high percentage of homozygous female rats died within a postpartum period of one month. This fact forced us to save females for reproductive purposes and they were not used for phenotype characterization.

### 2.2. Plasma Lipid and Lipoprotein Profiles of Male Cyp7b1-Deficient Rats Fed Regular Chow

Since mice lacking CYP7B1 had not shown any change regarding plasma lipids [6], rat plasma lipid levels were analyzed at two fasting periods. Following a short period of 4 h (Supplementary Table S3), no significant change was observed for total and HDL cholesterol, triglyceride, and APOA1 levels. However, APOA4 was found to be significantly decreased in mutant rats. In rats that underwent an overnight fast (Table 1), no significant change was observed in total and HDL cholesterol, nor in any other clinical chemistries except significant increases in plasma triglycerides and VLDL cholesterol in *Cyp7b1*-deficient rats. When plasma lipoproteins were prepared by FPLC and analyzed, the APOA1 profile shifted to smaller particles and increases in APOA4 in both HDL and LDL size fractions were noted (Supplementary Figure S2A,B). Cholesterol and phosphatidylcholine profiles also supported the slight increase in VLDL (Supplementary Figure S2C,D).

### 2.3. Characterization of Secreted Plasma Lipids and Lipoprotein Profiles of Rats Lacking CYP7B1

**Figure 1:**
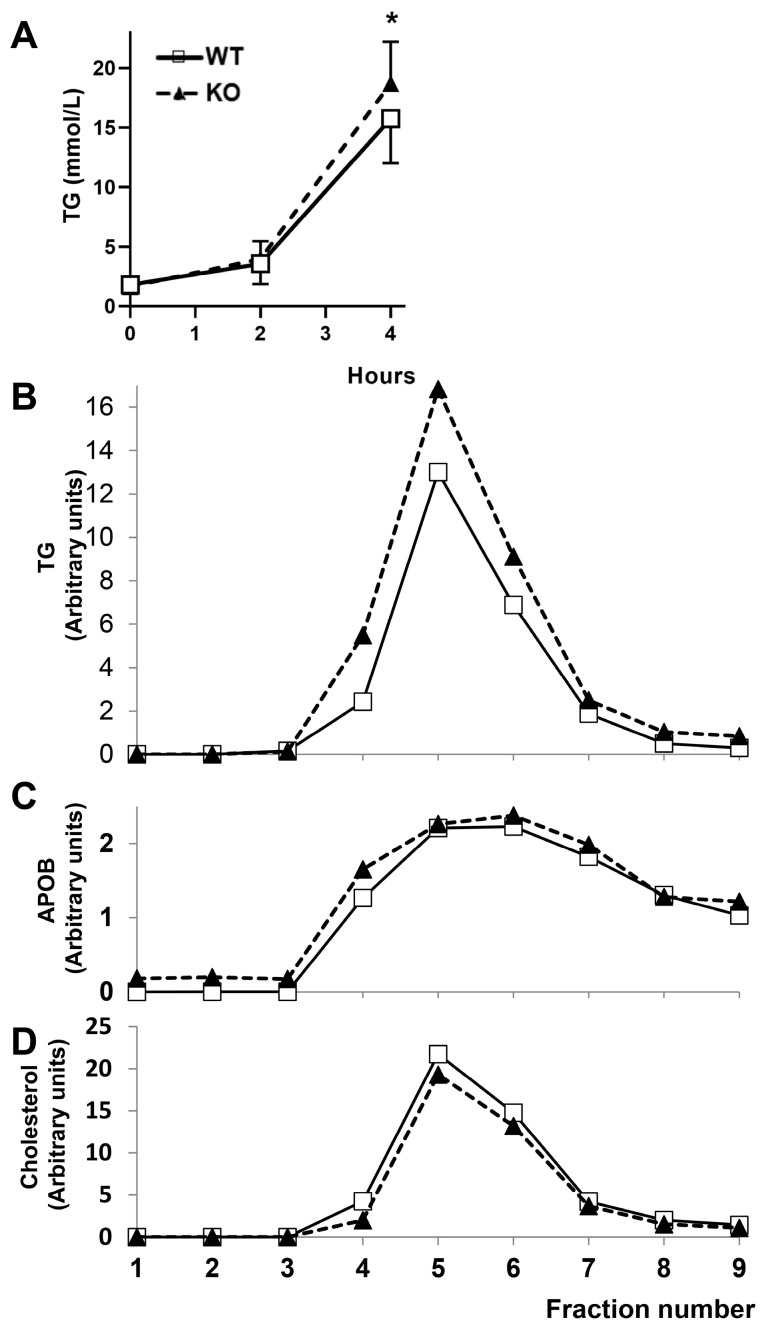
**Triglyceride secretion rate and analyses of secreted lipoproteins following tyloxapol administration in rats**. (**A**) Rate of plasma triglyceride concentration measured after a 16 h fast followed by intraperitoneal injection of tyloxapol (700 mg per kg body weight) in male wild-type and homozygous *Cyp7b1*-deficient rats (n = 11 per group). * *p* < 0.05 according to Mann–Whitney’s U-test. FPLC separation of plasma lipoproteins from both groups of rats four hours after tyloxapol injection and their APOB contents are reflected in panel (**C**). Their triglyceride and cholesterol contents are depicted in (**B**,**D**), respectively. Open squares correspond to wild-type and black triangles to *Cyp7b1*-deficient rats.

To characterize the observed increase in plasma triglyceride after prolonged fasting, an experiment was carried out to study secreted lipids after an overnight fast by blocking metabolism using tyloxapol. As shown in Figure 1A, a significant increase in secreted triglycerides was noted in rats lacking CYP7B1 four hours after tyloxapol administration. The increase in triglycerides was vehicled by VLDL (Figure 1B). No changes were observed for VLDL, APOB, and cholesterol (Figure 1C,D). These results point to abnormal secretion of VLDL in terms of TG composition rather than the number of particles.

### 2.4. Mechanisms of Increased Hepatic Secretion of VLDL in Rats Lacking CYP7B1

**Figure 2:**
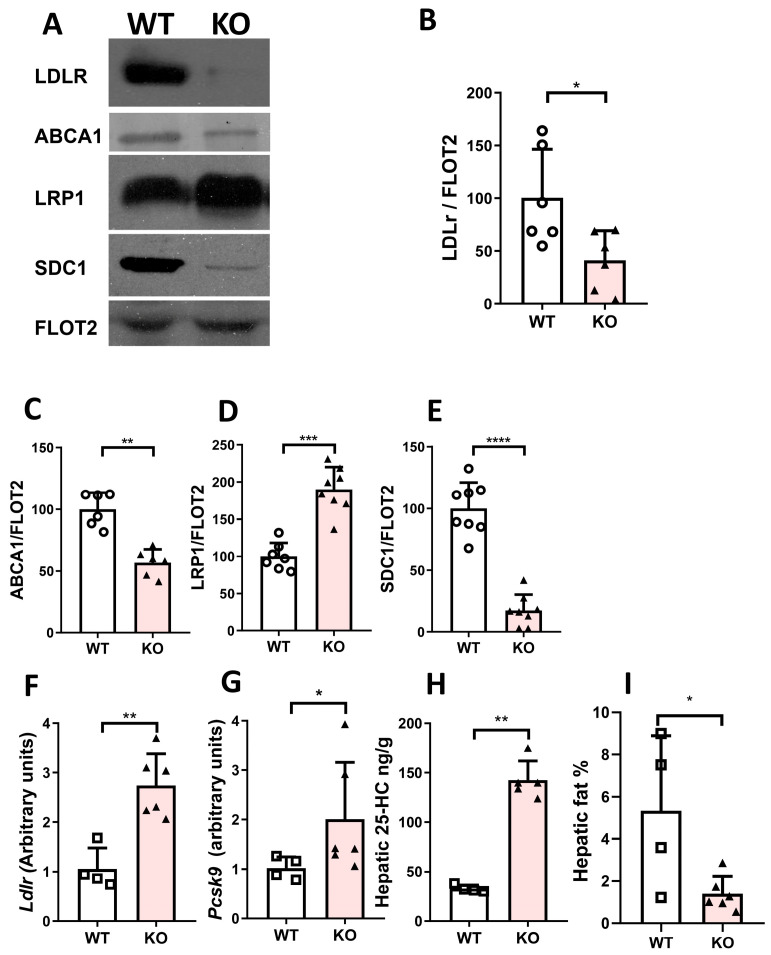
**Hepatic analyses of rats after 16 h of fasting.** (**A**) Representative Western blot analysis of plasma membrane proteins related to lipoprotein metabolism. Hepatic plasma membrane contents of (**B**) low-density lipoprotein receptor (LDLR); (**C**) ATP-binding cassette subfamily A member 1 (ABCA1); (**D**) low-density lipoprotein receptor-related protein 1 (LRP1); and (**E**) syndecan 1 (SDC1). Results of Western blot analyses were normalized to the data of flotillin 2 (FLOT2) as loading control. (**F**) *Ldlr* mRNA and (**G**) *Pcsk9* mRNA expressions. Values are reported as relative arbitrary unit (AU) levels obtained by RT-qPCR and normalized to *Rn18s.* (**H**) Hepatic levels of 25-hydroxycholesterol. (**I**) Morphometric changes in lipid droplet area expressed as a percentage of the total liver section. Data are shown as means ± SD with their individual values of 4 WT and 6 homozygous *Cyp7b1*-deficient (KO) rats. Statistical analysis was performed using the Mann–Whitney U-test. * *p* < 0.05, ** *p* < 0.02, *** *p* < 0.01, and **** *p* < 0.001 vs. control.

To investigate potential mechanisms responsible for the observed changes, hepatic plasma membranes were prepared and proteins involved in lipoprotein metabolism (ABCA1, LDLR, LRP1, and SDC1) analyzed. As shown in Figure 2A–C,E, *Cyp7b1*-deficient rats showed decreased hepatic plasma membrane contents of low-density lipoprotein receptor (LDLR, Figure 2B) and ATP-binding cassette subfamily A member 1 (ABCA1, Figure 2C) and syndecan 1 (SDC1, Figure 2E). However, those of low-density lipoprotein receptor-related protein 1 (LRP1, Figure 2D) were increased in rats with the inactivated *Cyp7b1* gene. Nevertheless, when the mRNA of *Ldlr* was analyzed, an increased expression was observed (Figure 2F). To understand this discrepancy between LDLR protein and its mRNA, expressions of *Pcsk9* were assayed and a significant increase was observed in *Cyp7b1*-knockout rats (Figure 2G). A significant increase in the content of 25-OH-cholesterol was also observed in the liver of *Cy7b1*-deficient rats (Figure 2H). The area occupied by lipid droplets decreased in the mutant rats (Figure 2I). No changes were observed in mRNA levels of microsomal transfer protein (*Mtp*), *Cideb*, and *Cidec* as proteins involved in the triglyceride loading of VLDL.

### 2.5. Response to a Fat Gavage

**Figure 3:**
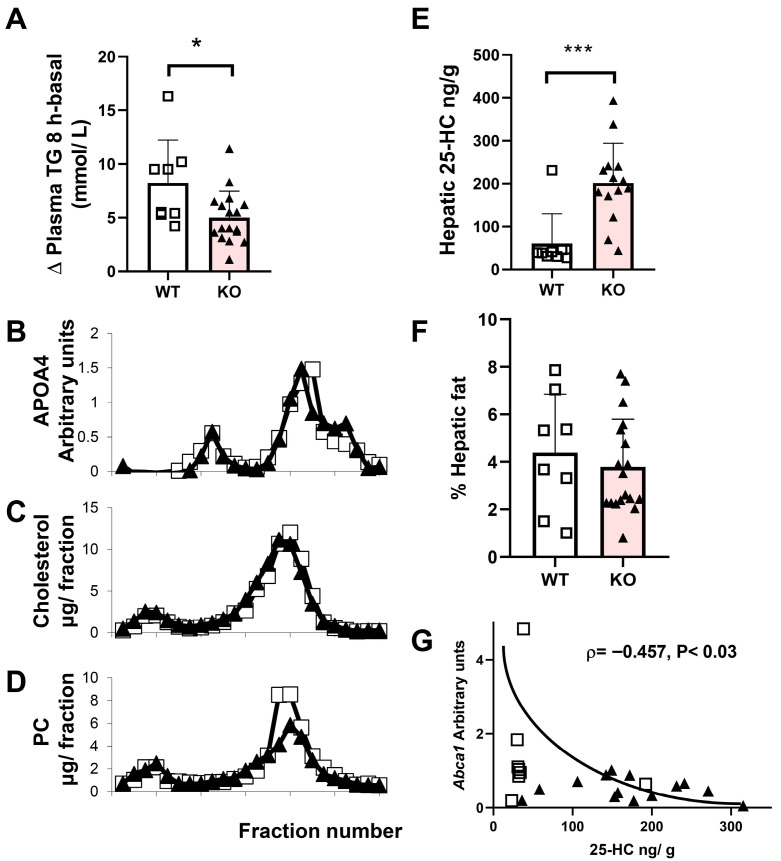
**Postprandial analyses of male rats eight hours after receiving a fat bolus**. (**A**) 16 h fasted male rats received an intragastrical administration of 5 mL of virgin olive oil (16 mL kg^−1^). Differential values of plasma TG eight hours after the oral gavage minus those from basal values are shown as individual, mean, and standard deviation values for wild-type (WT, n = 8) and homozygous KO (n = 17). Statistical analysis was performed using the Mann–Whitney U test. * *p* < 0.05 and *** *p* < 0.001 vs. control. Plasma lipoproteins were separated by FPLC and their APOA4 contents are reflected in panel (**B**). Their cholesterol and phosphatidylcholine (PC) contents are depicted in (**C**,**D**), respectively. Open squares correspond to wild-type and black triangles to homozygous *Cyp7b1*-deficient rats. (**E**) Hepatic levels of 25-hydroxycholesterol of rats sacrificed eight hours after the oral gavage. (**F**) Morphometric changes in the lipid droplet area expressed as a percentage of the total liver section. (**G**) Association between hepatic *Abca1* expression and levels of 25-hydroxycholesterol. Spearman’s ρ coefficient and significance are shown.

To explore the contribution of the intestine to the plasma triglycerides, the effects of acute fat loading were investigated in males, and plasma triglyceride levels eight hours after loading are shown in Figure 3. The lack of CYP7B1 decreases the postprandial hypertriglyceridemia response (Figure 3A) without inducing changes in APOA4 (Figure 3B) or total and HDL cholesterol (Figure 3C). Interestingly, HDL phosphatidylcholine was slightly decreased in mutant rats (Figure 3D). These HDL changes were not accompanied by APOA1 changes. When the hepatic expression of genes associated with HDL metabolism was studied (Supplementary Table S4), no significant change was observed, with the exception of a significant decrease in *Abca1*. Hepatic levels of 25-HC were more pronounced in this postprandial experimental setting than in the fasting state and the *Cyp7b1*-deficient rats showed significantly elevated values (Figure 3E). The hepatic area occupied by lipid droplets was not different between the two groups of rats (Figure 3F). A significant inverse association was observed between hepatic levels of 25-HC and hepatic *Abca1* mRNA levels (Figure 3G). No changes were observed in duodenal *Abca1* and *Abcg4* expressions.

### 2.6. Response to Oral Glucose Tolerance Tests in Rats

Fasting blood glucose levels (at 0 min after dosing) were comparable between the two groups. The OGTT showed that the blood glucose concentration peaked at approximately 30 min and then returned to the basal level within each experimental group (Supplementary Figure S3A). The AUC value calculated from Supplementary Figure S3A was also not altered by the absence of CYP7B1 (Supplementary Figure S3B). These results suggest that this gene inactivation did not affect glucose tolerance.

### 2.7. Search for Hepatocyte-Specific Targets of Cyp7b1 Deficiency in Rats

**Figure 4:**
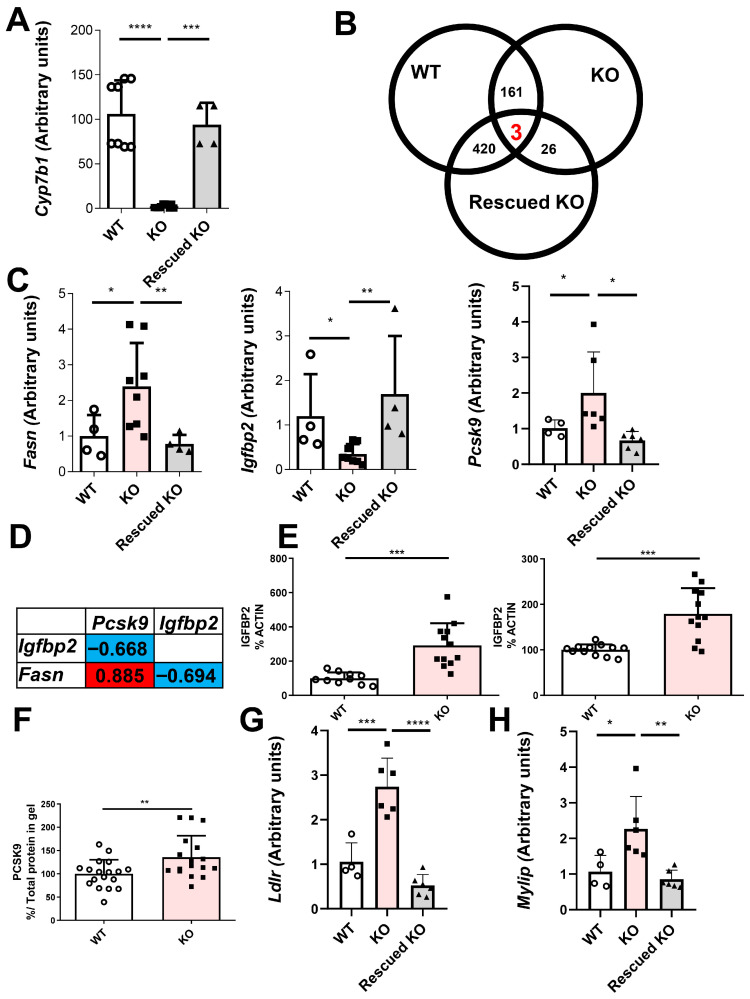
**Differentially expressed genes according to *Cyp7b1* deficiency in rats.** (**A**) Hepatic *Cyp7b1* mRNA expression in WT (n = 8), homozygous *Cyp7b1*-deficient (KO, n = 10), and homozygous *Cyp7b1*-deficient (n = 4) rats rescued by hydrodynamic expression of pLIVE-*Cyp7b1* (rescued KO) using RT-qPCR. (**B**) Venn diagram analysis showing the significant transcripts (false discovery rate < 0.01) among the different groups using RNAseq from the livers. (**C**) Hepatic expression of *Fasn*, *Igfbp2*, and *Pcsk9* normalized to *Rn18s* using RT-qPCR. (**D**) Significant association among gene expressions. Spearman’s ρ correlation coefficients are shown. (**E**) Western blot analysis of hepatic protein expressions of the non-glycosylated IGFBP2 isoform of 36 kDa normalized to ACTIN (**left**) and of the glycosylated IGFBP2 isoform of 50 kDa normalized to ACTIN (**right**). (**F**) Plasma protein content of PSK9 (74 kDa) normalized to total protein loaded into the gel. Data are shown as individual, means ± SD. Statistical analysis was performed using the Mann–Whitney U-test. ** *p* < 0.02 and *** *p* < 0.01 vs. control. (**G**) *Ldlr* and (**H**) *Mylip* expressions normalized to *Rn18s* using RT-qPCR. Data are means ± SD of arbitrary units. Statistical analysis was performed using one-way ANOVA and Tukey’s post hoc test. * *p* < 0.05, ** *p* < 0.02, *** *p* < 0.001, and **** *p* < 0.0001.

To address this issue, transcriptomic analyses were performed by RNAseq of hepatic mRNA from control, *Cyp7b1*-deficient, and *Cyp7b1*-deficient rats rescued by hydrodynamic expression of pLIVE-*Cyp7b1*. Prior to sequencing, the reversion of the phenotype by plasmid administration was analyzed by RT-qPCR. As shown in Figure 4A, this strategy rescued the hepatic *Cyp7b1* mRNA expression of *Cyp7b1*-deficient rats injected with the plasmid. A similar trend was observed for hepatic 25-HC, although there was a wide range of responses (33–143 ng/g related to the expression of Cyp7b1 mRNA reached). As shown by the Venn diagram of RNAseq changes, three gene expressions (*Fasn*, *Igfbp2*, and *Pcsk9*) were altered by CYP7B1 absence and rescued by pLIVE-*Cyp7b1* administration (Figure 4B). Quantitative PCR of these expressions confirmed the RNAseq results (Figure 4C). The absence of CYP7B1 increased the expression of *Fasn* and *Pcsk9* and decreased that of *Igfbp2*. The expressions of *Fasn* and *Pcsk9* were positively associated and both were inversely associated with that of *Igfbp2* (Figure 4D).

When the hepatic protein content of IGFBP2 was determined, an increase in both isoforms was observed in *Cyp7b1*-deficient rats (Figure 4E). Plasma levels of PCSK9 were also increased in the latter group (Figure 4F). Although an RNAseq procedure counting all transcripts of the genes *Ldlr* (12 for this gene, according to Gtex, https://gtexportal.org/home/gene/LDLR#geneExpression, accessed on 3 December 2025) and *Mylip* (2 for this gene) apparently showed no differences in the *Cyp7b1*-deficient rats, an RT-qPCR evaluating the complete transcripts showed a significant increase in both hepatic mRNA expressions by gene inactivation, and these changes were rescued by pLIVE-*Cyp7b1* administration (Figure 4G,H).

### 2.8. Characterization of a Stable Human CYP7B1-Knockout HepG2 Cell Line

**Figure 5:**
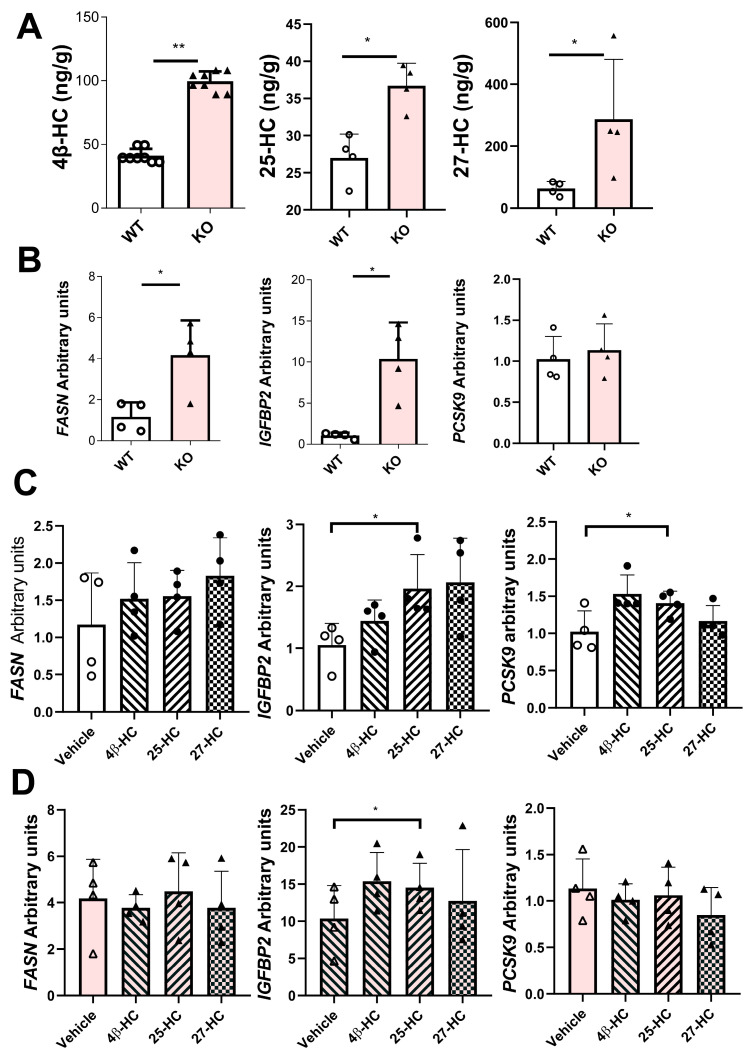
**Characterization of a stable human *CYP7B1*-knockout HepG2 cell line.** (**A**) Levels of oxysterols: 4β-hydroxycholesterol, 25-hydroxycholesterol, and 27-hydroxycholesterol. (**B**) Gene expressions of *FASN*, *IGFBP2*, and *PCSK9* normalized to *PPIB* in wild-type (WT) and *CYP7B1*-knockout (KO) HepG2 cells. (**C**) *FASN*, *IGFBP2*, and *PCSK9* gene expressions of wild-type HepG2 cells incubated with 250 nM 4β-hydroxycholesterol (4β-HC), 25-hydroxycholesterol (25-HC), or 27-hydroxycholesterol (27-HC) for 24 h. (**D**) Gene expressions of *CYP7B1*-KO HepG2 cells incubated with 250 nM 4β-hydroxycholesterol (4β-HC), 25-hydroxycholesterol (25-HC), or 27-hydroxycholesterol (27-HC) for 24 h. Data are shown as individual, means ± SD of four replicates except for 4β-hydroxycholesterol (eight replicates). Statistical analysis was performed using one-way ANOVA and Tukey’s post hoc test or Mann–Whitney’s U test. * *p* < 0.05 and ** *p* < 0.02.

As shown in Figure 5A, levels of 4β-hydroxycholesterol, 25-hydroxycholesterol, and 27-hydroxycholesterol were significantly increased in the cells with the inactivated *CYP7B1* gene. Its gene inactivation resulted in significantly increased expressions of *FASN* and *IGFBP2* and no change in that of *PCSK9* (Figure 5B). To delineate which of the three metabolites, 4β-hydroxycholesterol, 25-hydroxycholesterol, and 27-hydroxycholesterol, could be responsible for the observed changes in *FASN* and *IGFBP2*, HepG2 cells were incubated in vitro with each compound at 250 nM. The results showed that only 25-hydroxycholesterol significantly increased the expression of *IGFBP2* and *PCSK9* (Figure 5C). The former increase was also observed in HepG2 with the inactivated *CYP7B1* gene in the presence of 25-hydroxycholesterol (Figure 5D).

## 3. Discussion

Disruption of the *Cyp7b1* gene in rat zygotes produced rats lacking this gene expression. When male rats were characterized, they showed increased hepatic levels of 25-HC and plasma hypertriglyceridemia after overnight fasting, which was in agreement with abnormal secretion of VLDL in terms of TG composition rather than number of particles observed in the tyloxapol experiment and the decreased hepatic area occupied by lipid droplets in the mutant rats. In contrast, the plasma triglyceridemic response to a fat gavage was decreased in homozygotes lacking *Cyp7b1*. In this condition, *Cyp7b1*-deficient rats showed a decrease in HDL phosphatidylcholine accompanied by significantly decreased hepatic *Abca1* mRNA expression. Hepatic levels of 25-HC were more pronounced in this postprandial setting. Using RNAseq, the absence of CYP7B1 in rats resulted in increased expressions of *Fasn* and *Pcsk9* and decreased that of *Igfbp2.* When the hepatic protein contents of IGFBP2 were assayed, an increase in both isoforms was observed in *Cyp7b1*-deficient rats, accompanied by a normal glucose tolerance test. Plasma levels of PCSK9 were also increased in this group. A HepG2 cell line with an inactivated *CYP7B1* gene showed increased levels of 4β-HC, 25-HC, and 27-HC and significantly increased gene expressions of *FASN* and *IGFBP2* and no change in that of *PCSK9*. These results point to a role of CYP7B1 in the control of hepatic IGFBP2 and VLDL-TG without changes in a glucose oral tolerance test, suggesting a prediabetes state exerted through different hydroxycholesterol metabolites and transcriptional or translational mechanisms depending on the species.

The absence of CYP7B1 was found not to exert any effect on plasma triglycerides after a short fast interval of four hours, in agreement with data of knockout mice [6]. However, overnight fasting induced a significant increase in this parameter. Since the liver is the organ that delivers plasma triglycerides in this condition, its secretion of VLDL-TG was increased when blocking lipoprotein catabolism using tyloxapol. In the present model, normalization cannot take place by reuptake using LDLr since its presence in the hepatic plasma membrane was significantly decreased (Figure 2A,B). As in heterozygous mice lacking LDLr [27], no hypercholesterolemia was observed. An increase in plasma VLDL cholesterol (Table 1) and PC (Supplementary Figure S2D) was observed, suggesting that rats, similarly to mice, are dependent on APOE since they secrete VLDL-APOB48 from the liver [23]. APOE is a ligand for LRP1 [28]; considering the observed LRP1 increase in the hepatic plasma membrane (Figure 2A,D), a compensatory mechanism of VLDL recapture could exist and, for this reason, hypercholesterolemia following a long period of fasting was not observed.

Increased secretion of VLDL has been proposed as a mechanism of insulin resistance with increased reuptake to normalize LDL cholesterol levels [29]. The reduction in *Cyp7b1* expression was found in several genetic models of insulin resistance [4]. In fact, CYP7B1 loss has been proposed as an early alteration in the course of the disease and may be related to the progression from insulin resistance to type 2-like diabetes [16]. The present model provides further support to these statements and allows the establishment of a pathway in the sense that the absence of CYP7B1 increases 25-HC (Figure 2H) together with hepatic IGFBP2 (Figure 4E). The latter has been linked to increased production of apoB-48 containing chylomicrons [30]. Hepatocyte-specific ABCA1 knockout mice displayed triglyceride-enriched VLDL [31]. This mechanism may take place by CYP7B1 deficiency as reflected by its decrease in the plasma membrane (Figure 2C). The decreased syndecan 1 (Figure 2F) could also contribute to the increase in VLDL-TG due to its role in the clearance of these lipoproteins [32]. All these results reinforce the failure of CYP7B1 as a preliminary event in the cascade conducive to diabetes since this pathology was not observed regarding glucose levels and glucose tolerance tests (Supplementary Figure S3), but its perturbation compromises all lipoprotein receptors and translates into an increased VLDL-TG secretion rate as a preliminary event.

The increase in plasma PCSK9 (Figure 4F) could explain the decreased LDLR on the plasma membrane surface despite the increased *Ldlr* mRNA levels (Figure 4G) by blocking LDLR receptor recycling [29]. The PCSK9 increase was due to enhanced mRNA expression induced by CYP7B1 deficiency and the presence of increased hepatic 25-HC levels in rats.

ABCA1 is a lipid transporter present at the plasma membrane transferring cholesterol and phospholipids to lipid-poor or lipid-free APOA1 particles, thus generating pre-ß HDL [20]. It selectively transfers phosphatidylcholine, facilitating the preloading of APOA1 with this phospholipid [33,34]. Hepatic inactivation of ABCA1 in hepatocytes showed reduced HDL cholesterol [35]. The observed decrease in hepatic plasma membrane ABCA1 content (Figure 2C) may contribute to the smaller size of APOA1- and APOA4-containing particles and the increase in phosphatidylcholine in small HDL (Supplementary Figure S2A,B,D). The absence of changes in HDL cholesterol suggests a compensatory mechanism involving the removal of cholesterol from peripheral cells [33] since heterozygous mice lacking one copy of ABCA1 in the liver also showed reduced HDL cholesterol [35]. The present model evidences that the regulation of ABCA1 is dependent on 25-HC, but the effect may change according to the model studied.

In mice, elevated levels of 25-HC and 27-HC accumulated in CYP7B1 deficiency [6]. In rats, only the accumulation of 25-hydroxycholesterol was clearly observed (Figure 2H and Figure 3E). However, in HepG2, the inactivation of this gene resulted in increases in 4β-HC, 25-HC, and 27-HC (Figure 5A). This genetic modification questions the theoretical prediction of Cuie et al. [36], proposing 25-HC as the preferred substrate for the human enzyme. However, the metabolite influencing the studied parameters was 25-HC. Overall, these results indicate that the enzyme uses a wide range of substrates depending on the species, but 25-HC seems to be the most effective.

*Cyp7b1*-deficient rats are resistant to postprandial hypertriglyceridemia, a common feature with mice expressing human lipoprotein lipase [37] and *Apoc3*-deficient mice [38]. Contrary to these mice, these rats showed normal triglyceridemia in short periods of fasting, which is indicative of proper action of both the enzyme and inhibitor. Moreover, after prolonged fasting, an increase in plasma triglyceridemia was found, thus rejecting involvement of both proteins in the postprandial finding. In this setting, APOA4 levels and cholesterol were not modified, but phosphatidylcholine was particularly decreased and hepatic levels of 25-HC more pronounced than in the fasting state. These observations indicate a difficulty in intestinal absorption or packaging of triglycerides into chylomicrons and phosphatidylcholine in HDL, although peripheral actions of other lipase enzymes cannot be ruled out. These findings indicate that CYP7B1 plays an important role in coordinating intestinal and hepatic responses in the postprandial state.

## 4. Materials and Methods

### 4.1. Inactivation of Cyp7b1 Gene

The novel rat utilized was developed and obtained from the PhysGen Program in Genomic Applications at the Medical College of Wisconsin. Basically, the DNA from the Sleeping Beauty transposon BART3 gene-trap system containing an inverted repeat/direct repeat of the transposon, a splicing acceptor, primers for PCR detection of the transposon concatemers, and primer for PCR detection of gene trapping by splicing acceptor was injected into the pronuclei of rat zygotes from the Fischer 344 inbred strain. Independently, transgenic rats expressing SB11 transposase under the control of human PGK2 promoter were also generated [39]. Both strains were crossed several times and their progeny screened to verify the gene disrupted. A male offspring with transposon randomly inserted into the first intron of the *Cyp7b1* gene (Baylor 3.4/rn4, Chr2: 1031654226) was selected and was predicted to truncate the nascent *Cyp7b1* transcript after the first exon (F344-Cyp7b1^Tn(sb-T2/Bart3)2.306Mcwi^). The presence of the insertion was confirmed with a three-primer PCR assay using the genome-specific primers flanking the mapped insertion site 306_F: 5′-AAACATCACCTTCTGCAGAGGAC-3′ and 306_R: 5′-CCCTATTTGTATCTTGCTCAGCTTT-3′ and a BART3 transposon-specific primer 5′-CCTAACTGACTTGCCAAAAC-3′. This male was backcrossed to the Fisher 344 strain and heterozygous carriers were intercrossed and genotyped, using these primers, to generate a colony of rats for experiments.

### 4.2. Rats and Diets

Homozygous rats for the modified allele were generated by subsequent mating of heterozygotes. Rats were bred at the *Servicio de Experimentación Animal* (University of Zaragoza) and they were used for experiments when weighed 250–300 g and wre aged 2 months. Male rats, housed as 3–4 per cage, were maintained at 20 °C with a 12 h light-dark cycle, allowed ad libitum access to water and a chow diet for 1 month, and fasted for 4 or 16 h before experiments. The chow diet provided, expressed as energy, contained 15% proteins, 75% carbohydrates, and 10% lipids from soybean oil (TD 8604, Harlan S.A., Barcelona, Spain). All the procedures were performed observing criteria from the European Union guidelines for the handling and care of laboratory animals in research (Directive 2010/63/UE) and in accordance with ARRIVE guidelines, and the protocols were approved by the Ethics Committee for Animal Research of the University of Zaragoza (PI43/15).

### 4.3. Plasma Analyses

At the moment of sacrifice, fasted rats were anesthetized in a CO_2_ chamber and blood was drawn from their hearts. Blood was collected in tubes containing 1 g/L sodium EDTA. Organs were removed, weighed, and quickly frozen in liquid N_2_ until processed. An aliquot of tissue was kept in buffered formaldehyde. Plasma was separated by centrifugation of blood for 10 min at 8000× *g* at 4 °C. Plasma analytes were measured using commercial enzymatic methods using Infinity™ commercial kits (Thermo Scientific, Waltham, MA, USA). HDL cholesterol was determined in the supernatant after precipitating the ApoB-containing particles with phosphotungstic acid—MnCl_2_ (Roche, Barcelona, Spain). Apolipoproteins (APOA1, APOA4, and APOB) were quantified by ELISA using specific polyclonal antibodies (Biodesign #K23001R, Saco, ME, Santa Cruz Biotechnology #SC-19036, Biodesign #K23000R, Saco, ME, USA), as previously described [40]. Plasma lipoprotein profiles were determined in 100 μL of pooled plasma samples from each group by fast protein liquid chromatography (FPLC) gel filtration [41] using a Superose 6B column (Cytiva, Barcelona, Spain), and their cholesterol and phosphatidylcholine in each fraction were measured using the published procedures [42]. Biochemistry parameters (albumin, bilirubin, ALAT, ASAT, alkaline phosphatase, gamma-glutamyl transpeptidase, and glucose) were analyzed at the Clinical Laboratory of the *Hospital Clínico Universitario Lozano Blesa* (Zaragoza, Spain).

### 4.4. Analysis of Oxysterols by Liquid Chromatography/Mass Spectrometry (LC/MS) in the Liver and HepG2 Cell Line

Rat liver tissue extracts (200 mg) spiked with 60 µL of 100 µg/g cholesterol—26,26,26,27,27,27-D6 internal standard (Sigma-Aldrich, Madrid, Spain)—were processed using an ultra-high resolution ACQUITY UPLCTMH-Class liquid chromatograph from Waters (Milford, MA, USA) equipped with an ACQUITY UPLC^TM^BEH C18 2.1 mm × 150 mm column and coupled to an ACQUITY TQD^TM^ tandem quadrupole detector mass spectrometer from Waters. The operating conditions have been previously described [43], and the LOD was less than 54 pg mL^−1^ and the LOQ was lower than 171 pg mL^−1^ for all the assayed oxysterols. For quantification, 25-HC(d6) (internal standard 99%, CAS88247-69-2) and 27-HC(d6) (internal standard 99%, CAS 1246302-95-3), purchased from Avanti Polar Lipids (Alabaster, AL, USA), were used.

### 4.5. Histological Analyses

Tissues stored in neutral formaldehyde were included in paraffin. Sections (4 µm) were stained with hematoxylin and eosin. Captured images were used blindly for quantifying the extent of lipid droplets in each liver section and expressed as a percentage of the total liver area using Adobe Photoshop CS3 (Adobe, San Jose, CA, USA).

### 4.6. Fat Tolerance Test

Two-month-old male rats fasted for 16 h were anesthetized and a baseline fasting blood sample was obtained from the tail vein. Wild-type (n = 8) and *Cyp7b1*-KO (n = 18) rats were fed 5 mL of extra virgin olive oil (Aceites Toledo, Toledo, Spain) as a bolus, as described [42]. Eight hours after the gavage, rats were euthanized in a CO_2_ chamber and blood was drawn from their hearts. Blood was collected and organs were removed and quickly frozen in liquid N_2_.

### 4.7. Oral Glucose Tolerance Test

Two-month-old male rats fasted for 16 h were anesthetized and a baseline fasting blood sample was obtained from the tail vein. Each animal then received 2 g glucose/kg BW by gavage; this dose was chosen based on [44]. Blood samples were taken from the tail vein at 30, 60, 120, and 240 min after the glucose administration. The area under the curve (AUC) of blood glucose was calculated for each group.

### 4.8. Quantification of mRNA

RNA was isolated using TRI Reagent (Sigma). Contaminant DNA was removed by TURBO DNAse treatment from AMBION (Austin, TX, USA). RNA was quantified by absorbance at A_260/280_ (the A_260/280_ ratio was greater than 1.75). The integrity of the samples was verified by the 28S/18S ratio of ribosomal RNAs and the RNA integrity number (Agilent 2100 Bioanalyzer, Agilent, Santa Clara, CA, USA). Equal amounts of DNA-free RNA from each animal were used in RT-qPCR analyses. First-strand cDNA synthesis and the PCR reactions were performed using the SuperScript II Platinum Two-Step RT-qPCR Kit with SYBR Green (Invitrogen, Madrid, Spain), according to the manufacturer’s instructions. The primers used (Supplementary Tables S1 and S2) were designed with the National Center for Biotechnology Information (NCBI) primer design software and checked by BLAST (version 2.17.0) analysis (NCBI and Ensembl) to verify gene specificity and exon location. Real-time PCR reactions were performed in an ABI PRISM 7700 Sequence Detector (Applied Biosystems, Waltham, MA, USA) following the standard procedure. The relative amount of all mRNAs was calculated using the comparative 2^−ΔΔCq^ method. *Rn18s* was used to normalize gene expression changes [45].

### 4.9. Triglyceride Secretion Rate

Male wild-type (n = 4) and homozygous *Cyp7b1*-KO (n = 4) rats were fasted for 16 h and then intraperitoneally injected with Tyloxapol (Sigma) dissolved in PBS to provide a dose of 700 mg/kg per animal [42]. Blood samples were obtained from the tail vein at baseline and thereafter at intervals of 1 h for the following 4 h. Plasmas were used for triglyceride analyses and lipoprotein characterization by FPLC.

### 4.10. Microsomal and Plasma Membrane Preparations and Western Blot Analyses

The livers (200 mg) were processed as described [46]. The membranes were blocked and incubated with rabbit polyclonal antibodies against rat CYP7B1 (Everest Biotec #EB11484, Oxfordshire, UK), ABCA1 (Abcam #AB7360, Cambridge, UK), LDLR (Santa Cruz #SC-11824), LRP1 (Abcam #AB92544, Cambridge, UK), and SDC1 (Abcam #AB60199). Equal loadings were confirmed by using anti-HSC70 and anti-FLOT2 (Santa Cruz #SC-1059 and #SC-30750). The membranes were washed and incubated with anti-goat antibodies linked to horseradish peroxidase (Sigma HRP #A5420) or anti-rabbit HRP (Cytiva #NA934) and developed as described [47].

### 4.11. Hepatic Rescue of Cyp7b1-Deficient Rats

The open reading frame of the rat hepatic *Cyp7b1* transcript (NM_019138.1) was amplified by PCR using gene-specific primers (Fw: 5′-GTCGACATCGCTCACTACAGAGCCGCCA-3′ and Rv: 5′-CCGCGGGGCTGCATTTGGGGAGTACAGCA-3′) designed to add *SalI* and *SacII* restriction sites to the 5′ and 3′ ends, respectively. The PCR product was purified using the MinElute PCR Purification kit (Qiagen N.V., Venlo, The Netherlands), ligated into the pCR^®^II vector, transformed in One Shot^®^ INVαF’ cells (Invitrogen, Madrid, Spain), and sequenced. The full-length *Cyp7b1* cDNA product from pCR^®^II-*Cyp7b1*, excised by *SalI* and *SacII* restriction enzymes, was introduced into pLIVE™ (Mirus Bio, Madison, WI, USA). Confirmed by DNA sequencing, the pLIVE- *Cyp7b1* DNA was produced in large scale and purified using the Endofree Plasmid Purification kit (Qiagen). Hydrodynamic tail vein injection of *Cyp7b1*-deficient rats (n = 4) was performed by using 15 μg of the pLIVE-*Cyp7b1* plasmid diluted in a volume of PBS equivalent to 10% of the body weight [48,49]. WT (n = 4) and *Cyp7b1*-deficient (n = 8) rats were sham-injected with saline. The animals receiving chow diets were sacrificed 4 days after injection and their livers obtained.

### 4.12. RNAseq

To compare the liver gene expression profile modified by the absence of CYP7B1 in rats, total RNA was isolated from the liver of 4 WT, 8 *Cyp7b1*-KO, and 4 *Cyp7b1*-KO rescued by hydrodynamic injection of pLIVE-*Cyp7b1*. The RNA sequencing library was prepared with the TruSeq RNA Sample Preparation v2 Kit (Illumina, San Diego, CA, USA) to construct index-tagged cDNA. Libraries were sequenced on the Genome Analyzer IIx (Illumina) following the standard RNA sequencing protocol with the TruSeq SBS Kit v5. The number of reads obtained per sample was in the range of 25 to 41 million. Sequencing reads were pre-processed by the Cutadapt software tool (version 5.2) [50] to trim sequencing reads, eliminate Illumina adaptors, and discard reads that were shorter than 30 bp. The resulting reads were mapped against the rat transcriptome (Ensembl Rnor 6.0, release 82) and quantified using RSEM v1.17 [51]. Differential expression analyses were carried out using EdgeR (version 4.6.4) [52]. We thank the CNIC Genomics Unit (Madrid) for their help with these experiments. The dataset was deposited in the NCBI GEO public database with the number GSE267177.

### 4.13. HepG2 Incubations

The human hepatocyte cell line HepG2 was grown as described [53]. After one week of growth, the medium was removed and cells were washed with phosphate-buffered saline (PBS) prior to the addition of the serum-free media supplemented with 0.2% ethanol or 250 nM sterols dissolved in ethanol. After a 24 h incubation, the media were removed and cells were collected with TRI reagent solution (Ambion, Austin, TX, USA). RNA isolation and cDNA synthesis were carried out as described above.

### 4.14. Generation of a Stable CYP7B1 Knockout HepG2 Cell Line

The cell line was grown for one week. Then, the medium was withdrawn, and the cells were washed twice with PBS before being transfected with *CYP7B1* HDR and *CYP7B1* CRISPR/Cas9 KO plasmids (Santa Cruz Biotechnology) using lipofectamine 2000 (Thermo Fisher Scientific). The CYP7B1 CRISPR/Cas9 KO plasmid possesses gRNA sequences to generate double-stranded breaks specifically in a 5′ constitutive exon of *CYP7B1*. To provide the selection of constitutive knockout (KO) HepG2 cells, the CYP7B1 HDR recombined the *CYP7B1* gene containing a puromycin resistance gene. Puromycin-resistant HepG2-KO cells were selected after several rounds of puromycin incubations. CYP7B1 absence was confirmed by quantification of oxysterols.

### 4.15. Statistical Analysis

Results are expressed as means ± SD. Comparisons were made using one-way ANOVA and the Tukey–Kramer multiple comparison test (post hoc) when the distribution of the variables was normal. When the variables did not show such a distribution (according to the Shapiro–Wilk test) or failed to show homology of variance, comparisons were made using the Mann–Whitney U test using Prism 8 software for Windows (GraphPad, San Diego, CA, USA). Correlations among variables were sought using Spearman’s correlation tests. All calculations were performed using the Statistical Package for Social Sciences version 29 (IBM, Armonk, NY, USA). Significance was set at *p* ≤ 0.05.

## 5. Conclusions

**Figure 6:**
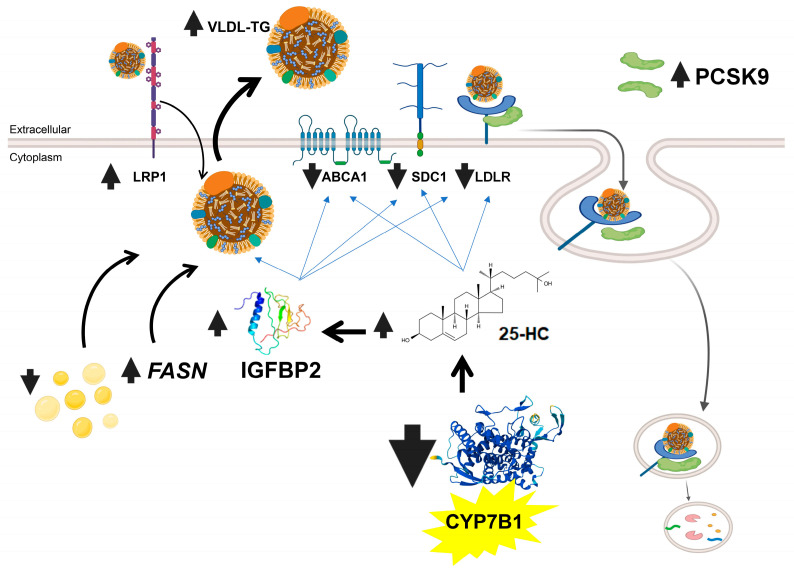
**Summary of phenotype of *Cyp7b1* deficiency in rats.** The absence of this enzyme increases levels of 25-HC and through this, broad metabolic control is exerted on FASN, IGFBP2, PCSK9, LDLR, SCD1, ABCA1, and LRP1. Created in Biorender.com, Osada (2025), https://app.biorender.com/illustrations/67ddc3c130709ae66c1676b6.

This work describes animals that are useful for investigating the influence of CYP7B1 on the fine tuning of physiological triglyceridemia in fasting and postprandial settings. Further studies on these rats may help link CYP7B1 to precocious changes in the development of insulin resistance, particularly regarding hepatic secretion of TG-enriched VLDL and IGFBP2 levels via oxysterols (Figure 6). The present study clearly shows that CYP7B1 modulates the release of these kinds of particles from the liver in the fasting state, influencing LRP1 and syndecan 1 and LDLr protein on the hepatocyte plasma membranes, the latter by increasing plasma PCSK9. It also plays an important role in the management of postprandial triglycerides. Overall, CYP7B1 plays a central role in the management of lipids in both rat liver and intestine subjected to physiological fasting or postprandial challenges.

## Figures and Tables

**Table 1:** Plasma parameters in homozygous male *Cyp7b1*-deficient rats.

	Wild-Type (n = 5)	*Homozygous* *Cyp7b1*-KO (n = 7)
Cholesterol (mg/dL)	115 ± 33	117 ± 26
Glucose (mg/dL)	263 ± 177	368 ± 99
HDL cholesterol (mg/dL)	114 ± 33	111 ± 24
VLDL cholesterol (mg/dL)	1 ± 0.4	5 ± 1 ***
Triglycerides (mg/dL)	118 ± 59	194 ± 26 *
Albumin (g/dL)	4.8 ± 0.2	5.0 ± 0.1
Bilirubin (mg/dL)	0.04 ± 0.01	0.04 ± 0.01
ALAT (IU/L)	53 ± 15	79 ± 27
ASAT (IU/L)	106 ± 31	102 ± 40
Alkaline phosphatase (IU/L)	0.3 ± 0.5	0.3 ± 0.5
Gamma-glutamyl transpeptidase (IU/L)	0.2 ± 0.8	0.2 ± 0.6
Urea (mg/dL)	0.4 ± 0.1	0.4 ± 0.1

Data are means ± standard deviation of 16 h fasted rats receiving a chow diet. Statistical analysis was performed using the Mann–Whitney U test. *** *p* < 0.001 and * *p* < 0.05 vs. wild-type.

## Data Availability

The dataset was deposited in the NCBI GEO public database with the number GSE267177.

## Supplementary Materials

The following supporting information can be downloaded at https://www.mdpi.com/article/10.3390/ijms262411994/s1.

## Abbreviations

The following abbreviations are used in this manuscript:

ALAT	alanine aminotransferase
ASAT	aspartate aminotransferase
25-HC	25-hydroxycholesterol
27-HC	27-hydroxycholesterol
HDL	High-density lipoprotein
LDLR	low-density lipoprotein receptor
LRP1	low-density lipoprotein receptor related protein 1
SDC1	syndecan 1
