# Supplementary material for: The Disruption of Cyp7b1 Controls IGFBP2 and Prediabetes Exerted Through Different Hydroxycholesterol Metabolites
Source: Int J Mol Sci. 2025 Dec 12;26(24):11994. doi: 10.3390/ijms262411994 (PMC12732954; doi:10.3390/ijms262411994)
Supplement: Supplementary file 1 [file ijms-26-11994-s001.zip › ijms-4006646-supplementary-Figures.pdf]

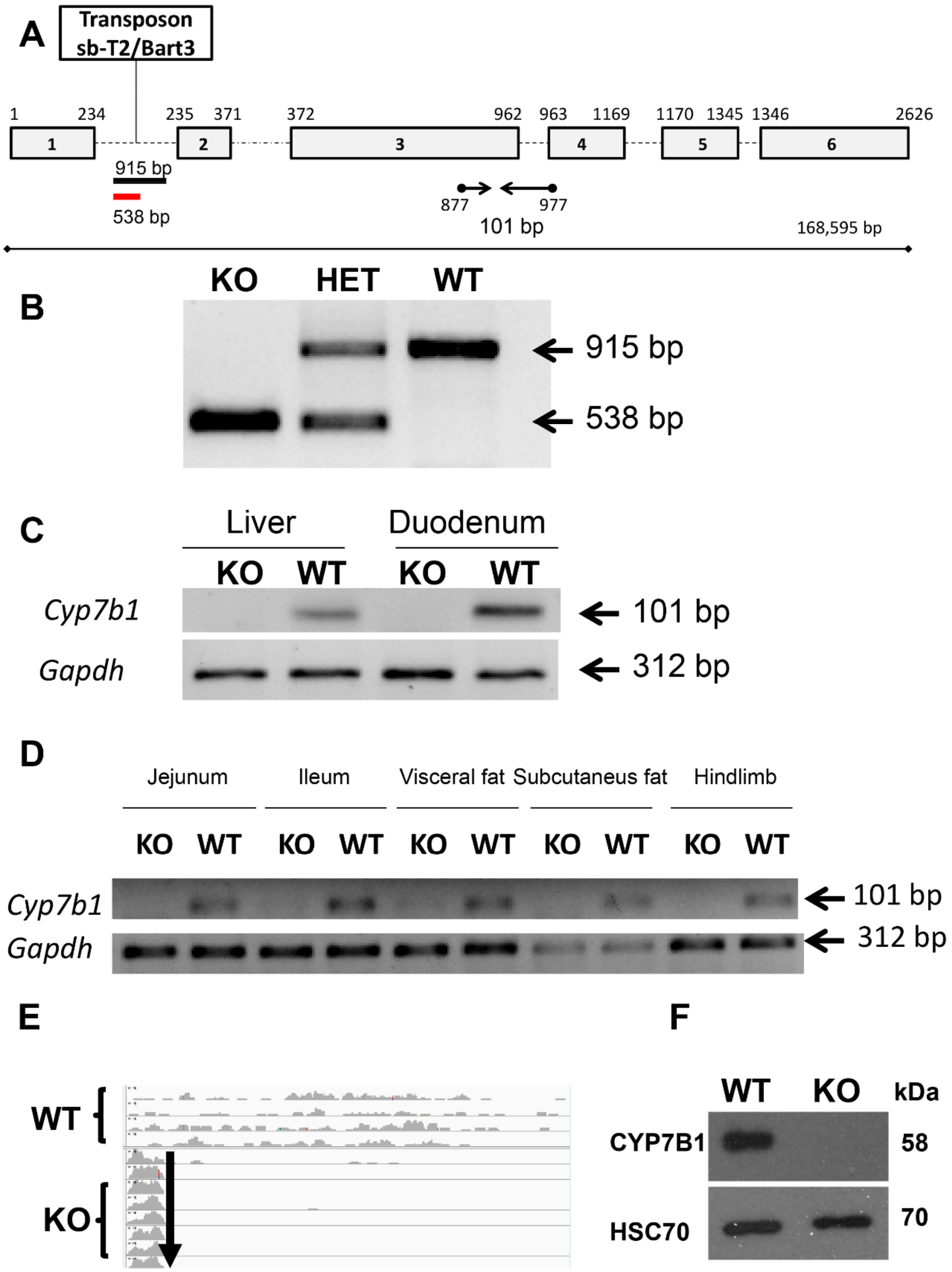

Supplementary Fig S1. Disruption of the rat *Cyp7b1* gene. A, schematic representation of the wild-type *Cyp7b1* gene showing the sizes of exons 1-6 and total length of the gene (https://www.ncbi.nlm.nih.gov/gene/25429), insertion of the transposon and location of primers used to genotype and confirm the mRNA expression. *bp*, base pair. B, PCR

genotyping of homozygous (KO) with the transposon inserted in both DNA copies, heterozygous (Het) and wild-type (WT) rats. Wild-type and mutated allele gave bands of 915 and 538 bp, respectively and revealed the insertion in the first intron. C and D, RT-PCR analysis of mRNA expression in several tissues from male homozygous mutant (KO) and wild-type (WT) rats. *Gapdh* was used as loading control. E, RNA sequencing of hepatic mRNA from four wild-type and eight transposon-inserted homozygous rats. Alignments of sequence readings in the different animals for the *Cyp7b1* gene according to Integrative Genome Viewer 2.8.2®. The downward arrow indicates the position where transcription was halted in KO rats. F, Western-blotting showing CYP7B1 protein wild-type and KO rat hepatic microsomal preparations.

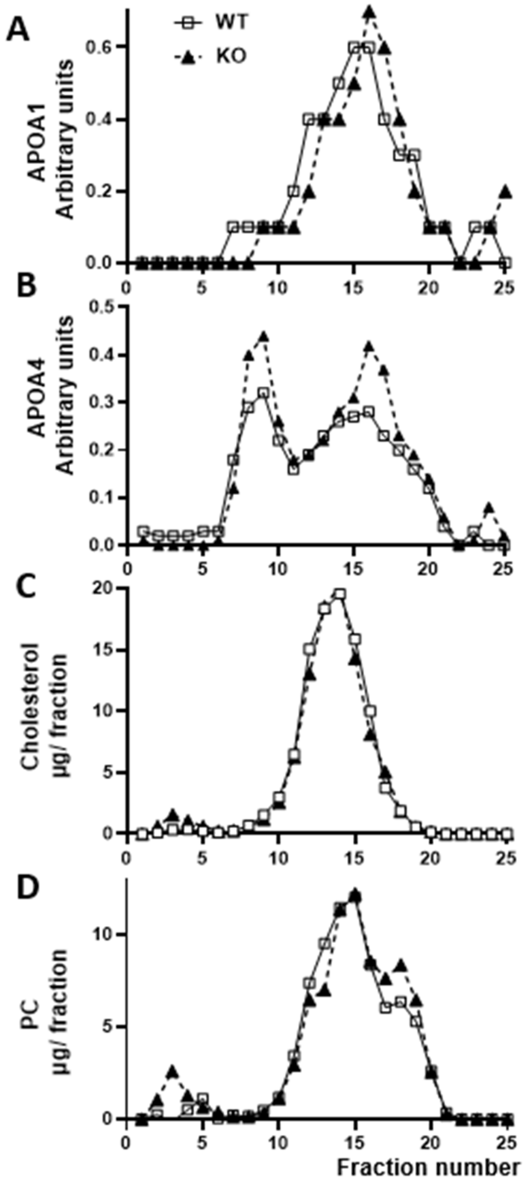

Supplementary Fig. S2. Characterization of male rat plasma lipoproteins. Plasma lipoproteins were separated by FPLC and their APOA1 and APOA4 contents are reflected in panels A and B, respectively. Their cholesterol, and phosphatidylcholine (PC) contents are depicted in C, and D, respectively. Open squares correspond to wild-type and black triangles to homozygous *Cyp7b1*-KO rats fasted for 16 hours.

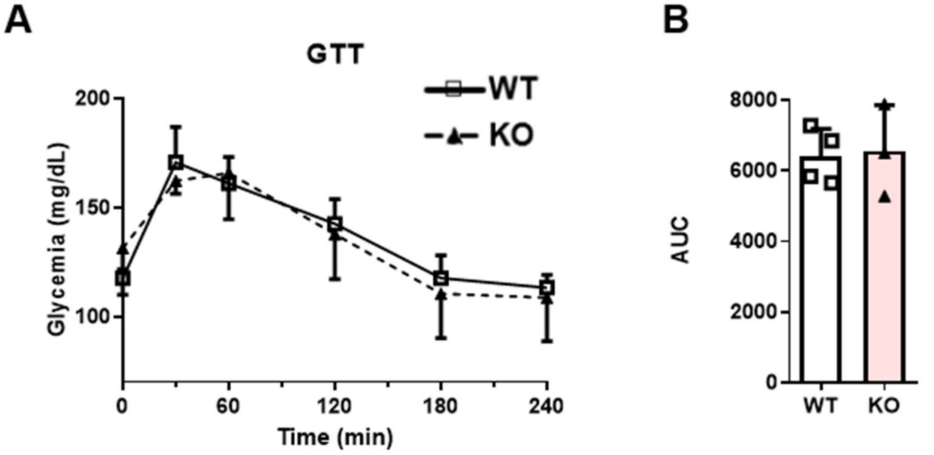

Supplementary Fig. S3. Oral glucose tolerance tests on male *Cyp7b1*-deficient rats. Glucose follow-up (A) of an oral glucose tolerance test (OGTT) in homozygous *Cyp7b1*-deficient (KO, n=3) and wild type rats (n=4). Area under the curve of OGTT (B) was calculated with curve fitting taking baseline for each condition. Data are means ± SD for each group.
