# Supplementary material for: The Disruption of Cyp7b1 Controls IGFBP2 and Prediabetes Exerted Through Different Hydroxycholesterol Metabolites
Source: Int J Mol Sci. 2025 Dec 12;26(24):11994. doi: 10.3390/ijms262411994 (PMC12732954; doi:10.3390/ijms262411994)
Supplement: Supplementary file 1 [file ijms-26-11994-s001.zip › ijms-4006646-supplementary-Tables.pdf]

Supplementary Table S1. Nucleotide sequence of rat primers used for RT-qPCR.

**Gene**	**Accession #**		**Sequence**	**Junction**	**[primer]**	**Efficiency**	**Amplicon length**
Abca1	NM_178095	forward	TCGGCTGGTATCGATTTCACA	exon 10/11	100 nM	96%	130
		reverse	GGTCCCAGTACCCATCCTTGAT				
Abcg1	NM_053502	forward	TCTGACCTTTCCCCTCGAGAT	exon 11/13	100 nM	100%	146
		reverse	AGTACACGATGCTGCAGTAGGC				
Apoa1	NM_012738	forward	GGCAGAGACTATGTGTCCCAGTTT	exon 3/4	100 nM	100%	91
		reverse	TTGAACCCAGAGTGTCCCAGTT				
Apoa1b	NM_001106440	forward	CGGCTATTGCCAAGGCTTAT	exon 1/2	100 nM	94%	123
		reverse	AAAGTTTGAGGTGTCGCGCA				
Apoa2	NM_013112	forward	GCCTAGAAGGAGCTTTGGTTCG	exon 1/2	100 nM	98%	141
		reverse	TGGCTTGGTTCTGAATCTCTGA				
Apoa4	NM_012737	forward	ACCCTCTTCCAGGACAAACTTG	exon 3	100 nM	96%	103
		reverse	CCTTGGTTAGATGTCCACTCAGTTG				
Apoc2	NM_001085352.1	forward	CATGTCCCTCAGTTTCTCGTC	exon 3/4	100 nM	99%	133
		reverse	CGGGCAGCTCAGCTCTGCTG				
Apoc3	NM_001271053.1	forward	CTCTGCCCGAGCTGATGA	exon 2/3	100 nM	95%	163
		reverse	CAGGGATTTGAAGCGATTGT				
Apoe	NM_138828	forward	CTTGTTTCGGAAGGAGCTGACT	exon 2	100 nM	96%	89
		reverse	AGGCATCCTGTCAGCAATGTG				
Apom	NM_019373.2	forward	TCAGAACTGAAGGGCGCCCA	exon 3/4	100 nM	96%	80

		reverse	CGTCTCTTTCAGCATGATTCC				
Cdig2	NM_153624.2	forward	GAATCCCACCTGCATCATTT	exon 8	100 nM	98%	170
		reverse	ACGAAAGAAAGGCCCAAAGT				
Cideb	NM_001108869.1	forward	ATGGCCTGCTAAGGTCAGTG	exon 1/3	100 nM	100%	191
		reverse	AAGTCTTCAGTCTCCACCGC				
Cidec	NM_001024333	forward	TCACTGTCCAGGCATGTAGCA	exon 2/3	100 nM	99%	127
		reverse	CCTTTGCGAACCTTCCGAT				
Fasn	NM_017332.2	forward	GCCTAACACCTCTGTGCAGT	exon 5/6	100 nM	101%	92
		reverse	GGCAATACCCGTTCCCTGAA				
Hprt	NM_012583	forward	TCCCAGCGTCGTGATTAGTGA	exon 1/3	100 nM	97%	152
		reverse	CCTTCATGACATCTCGAGCAAG				
Igfbp2	NM_013122.2	forward	GACGTTACGCTGTTACCCCA	exon 1/2	100 nM	96%	128
		reverse	GGTCATCCTCACTGTCTGCAA				
Lcat	NM_017024	forward	GGCTGTGCTACCGAAAGACAGA	exon 2/3	100 nM	97%	105
		reverse	GACAACCCTGGTGTTATCAATCCA				
LDLr	NM_175762.2	forward	GACTGCAAGGACAAGTCGGA	exon 4/5	100 nM	98%	168
		reverse	GCACTGGGTCACATTGATGC				
Lpl	NM_012598.2	forward	TTCCCTTCACCCTGCCGGAG	exon 7/8	100 nM	97%	129
		reverse	CTGACCAGCGGAAGTAGGAG				
Mylip	NM_001107344.2	forward	ACTGTCTCAACCAGGTGTGC	exon 1/2	100 nM	96%	151
		reverse	TTAAGCCGGTAAGGTGCCAG				

Pcsk9	NM_199253.2	forward	ATGAGCAGTGACCTGTTGGG	exon 2/3	100 nM	94%	155
		reverse	CTTCCGTCAGGGGAGCTATC				
Pla2g7	NM_001009353	forward	GCGTTTGTACTACCCAGCTCAAGA	exon 4/5	100 nM	99%	153
		reverse	TGCAGGAGTTGTCAGAGAACCA				
Pltp	NM_001168543	forward	GTTGAATGAGCGTATCTGGCGT	exon 14/16	100 nM	99%	101
		reverse	CAACAGTGACGAAGCCTGCAT				
Pon1	NM_032077	forward	GGACTGGTGTTGGCACTTTACA	exon 1/3	100 nM	98%	124
		reverse	CACCCGCTTCGATTCCTTTA				
Scarb1	NM_031541	forward	TCAAGAATGTCCGCATAGACCC	exon 1/2	100 nM	98%	128
		reverse	TTCTGGCCATTTAGGACCTCG				
Rn18s	X01117	forward	ACTCAACACGGGAAACCTCA	exon 5	100 nM	99%	114
		reverse	TCTTAGTTGGTGGAGCGATT				
Syt1	NM_001033680	forward	CAGAATAACCAGCTGTTGGTGG	exon 6/7	100 nM	96%	109
		reverse	TTTTGTCAGGCAGCAGGAAG				
Tbp	NM_001004198	forward	TAATCCCAAGCGGTTTGCTG	exon 4/6	100 nM	92%	111
		reverse	TTCTTCACTCTTGGCTCCTGTG				
Ubc	NM_017314	forward	ATCTAGAAAGAGCCCTTCTTGTGC	exon 3	100 nM	98%	51
		reverse	ACACCTCCCCATCAAACCC				

Supplementary Table S2. Nucleotide sequence of primers used for RT-qPCR in HepG2 cells.

Gene	Accession #		Sequence	Junction	[primer]	Efficiency	Amplicon [illegible]
FASN	NM_004104.5	forward	CAGAGCAGCCATGGAGGAG	exon 1/3	100 nM	97 %	144
		reverse	TAGAGCCCCGCCTTCCAG				
IGFBP2	NM_000597.3	forward	GAGCAGGTTGCAGACAATGG	exon 1/2	100 nM	98 %	160
		reverse	CAGTGACCTTCTCCCGGAAC				
PCSK9	NM_174936.4	forward	GACTACGAGGAGCTGGTGC	exon 1/2	100 nM	93 %	107
		reverse	CACGGATCCTTGGCGCAG				
PPIB	NM_000942.5	forward	GGAGATGGCACAGGAGGAA	exon 2	100 nM	100 %	71
		reverse	TAGTGCTTCAGCTTGAAGTTCTCAT				

Supplementary Table S3. Plasma lipid profiles in male rats deficient in *Cyp7b1*

	Male	
	Wild-type (n=20)	*Homozygous Cyp7b1*-KO (n=14)
Cholesterol (mg/dL)	136 ± 23	123 ± 27
HDL-cholesterol (mg/dL)	115 ± 19	104 ± 23
Triglycerides (mg/dL)	227 ± 96	227 ± 96
APOA1 (AU/ L)	62 ± 15	60 ± 9
APOA4 (AU/ L)	60 ± 8	51 ± 10*

Data are means ± standard deviation of 4-hour fasted rats receiving chow diet.

Statistical analysis to evaluate dietary response was done using the Mann Whitney's U test. * P< 0.05 vs wild-type

Supplementary Table S4. Hepatic postprandial gene expression in male rats following a 5 mL gavage according to genotype

	Wild-type (n=8)	*Homozygous Cyp7b1*-KO (n=18)
Abca1	1.0 ± 0.3	0.5 ± 0.4*
Abcg1	1.0 ± 0.1	0.9 ± 0.4
Apoa1	1.0 ± 0.2	0.9 ± 0.1
Apoa1bp	1.0 ± 0.2	1.0 ± 0.1
Apoa2	1.0 ± 0.1	0.9 ± 0.1
Apoa4	1.0 ± 0.1	0.9 ± 0.1
Apoc2	0.9 ± 0.3	0.7 ± 0.2
Apoc3	1.0 ± 0.1	0.8 ± 0.1
Apoe	1.0 ± 0.1	0.8 ± 0.1
Apom	1.0 ± 0.1	0.8 ± 0.1
A2m	1.0 ± 0.6	1.4 ± 1.5
Cdig2	1.0 ± 0.2	0.8 ± 0.1
Fabp4	1.2 ± 0.7	1.5 ± 1.2
Lcat	1.0 ± 0.4	0.8 ± 0.2
Lpl	1.0 ± 0.1	1.0 ± 0.1
Pltp	1.0 ± 0.1	1.0 ± 0.1
Pon1	1.0 ± 0.1	0.8 ± 0.1
Scarb1	0.9 ± 0.3	0.8 ± 0.2
Slc13a5	1.5 ± 1.0	1.5 ± 1.1

Values are means ± standard deviations of rats sacrificed eight hours after the oral gavage. Data represent arbitrary units obtained with the RT-qPCR normalized to *Rn18s*. Statistical analysis was done using the Mann Whitney's U test. *P<0.05 vs wild-type.
